# S100A16 promotes acute kidney injury by activating HRD1-induced ubiquitination and degradation of GSK3β and CK1α

**DOI:** 10.1007/s00018-022-04213-5

**Published:** 2022-03-12

**Authors:** Yifei Sun, Ya Fan, Zheng Wang, Min Li, Dongming Su, Yun Liu, Xiubin Liang

**Affiliations:** 1 Department of Pathophysiology, Nanjing Medical University, Nanjing, 211166 Jiangsu China; 2 Department of Pathology, Nanjing Medical University, Nanjing, 211166 China; 3 Department of Geratology, The First Affiliated Hospital of Nanjing Medical University, Nanjing, 210029 China

**Keywords:** S100A16, Acute kidney injury, Wnt/β-catenin, Ischemia–reperfusion injury, Renal interstitial fibroblasts

## Abstract

The pathogenesis of acute kidney injury (AKI) is associated with the activation of multiple signaling pathways, including Wnt/β-catenin signaling. However, the mechanism of Wnt/β-catenin pathway activation in renal interstitial fibroblasts during AKI is unclear. S100 calcium-binding protein A16 (S100A16), a new member of calcium-binding protein S100 family, is a multi-functional signaling factor involved in various pathogenies, including tumors, glycolipid metabolism disorder, and chronic kidney disease (CKD). We investigated the potential participation of S100A16 in Wnt/β-catenin pathway activation during AKI by subjecting wild-type (WT) and S100A16 knockout (S100A16^+/−^) mice to the ischemia–reperfusion injury (IRI), and revealed S100A16 upregulation in this model, in which knockout of S100A16 impeded the Wnt/β-catenin signaling pathway activation and recovered the expression of downstream hepatocyte growth factor (HGF). We also found that S100A16 was highly expressed in Platelet-derived growth factor receptor beta (PDGFRβ) positive renal fibroblasts in vivo. Consistently, in rat renal interstitial fibroblasts (NRK-49F cells), both hypoxia/reoxygenation and S100A16 overexpression exacerbated fibroblasts apoptosis and inhibited HGF secretion; whereas S100A16 knockdown or Wnt/β-catenin pathway inhibitor ICG-001 reversed these changes. Mechanistically, we showed that S100A16 promoted Wnt/β-catenin signaling activation via the ubiquitylation and degradation of β-catenin complex members, glycogen synthase kinase 3β (GSK3β) and casein kinase 1α (CK1α), mediated by E3 ubiquitin ligase, the HMG-CoA reductase degradation protein 1 (HRD1). Our study identified the S100A16 as a key regulator in the activation of Wnt/β-catenin signaling pathway in AKI.

**Supplementary Information:**

The online version contains supplementary material available at 10.1007/s00018-022-04213-5.

## Introduction

AKI is associated with high morbidity and mortality, claiming about 1.7 million lives worldwide each year [1]. AKI is clinically considered a rapid decline in renal function from a variety of causes, of which IRI is widely considered the most important cause [2]. Initially considered a self-healing disease, recent studies have shown that severe AKI leads to incomplete renal repair, persistent chronic inflammation, fibrosis progression, and eventually chronic organ loss [3, 4]. The long-term consequences of AKI include CKD and even serious end-stage renal disease (ESRD), both associated with a poor quality of life and high cost of care, thereby imposing a significant burden on the society [5, 6]. Therefore, understanding the pathogenesis of AKI, and developing prevention and treatment strategies are of the great significance for prolonging the lifespan of patients and reducing the societal burden.

Multiple signaling pathways contribute to the pathogenesis of AKI. In particular, the activation of Wnt/β-catenin pathway plays an irreplaceable role in severe AKI. Wnt/β-catenin signaling is an evolutionarily conserved developmental signaling pathway that is involved in organogenesis, tissue balance, and disease progression [7, 8]. In the adult kidney, Wnt signaling is usually silent [9, 10]. However, when the kidney is subjected to IRI, Wnt/β-catenin signaling is activated in the cells of the kidney [11, 12]. In the canonical Wnt/β-catenin signaling pathway, the transcriptional coactivator β-catenin is regulated by the β-catenin degradation complex, which consists of GSK3β, CK1α, adenomatous polyposis coli (APC), and scaffold protein (Axin) [13]. In the absence of Wnt (Wnt-off), β-catenin is phosphorylated by the β-catenin degradation complex. Phosphorylated β-catenin is recognized for proteasome degradation by the E3 ubiquitin ligase β-Trcp, and the loss of β-catenin subsequently inhibits the transcription of Wnt target genes in the nucleus. However, when the kidney is severely damaged, the Wnt ligands bind to the transmembrane frizzled protein receptor (FZD) and to the low-density lipoprotein receptor-related protein 5 or 6 (LRP5/6) in the Wnt-on (containing Wnt) state. These events lead to the destruction of the β-catenin degradation complex, thereby impairing the degradation of β-catenin in the cytoplasm. Subsequently, the stable and accumulated β-catenin is transferred to the nucleus, where it stimulates transcription of downstream target genes [14, 15]. Therefore, the activation of Wnt/β-catenin signaling pathway is a key feature in the occurrence and development of AKI. Recent studies have demonstrated that renal interstitial fibroblast plays an important role in the activation of Wnt/β-catenin signaling in short-term IRI induction (24 h after IRI surgery). Renal interstitial fibroblasts can secrete a range of growth factors, including HGF, to regulate kidney growth and repair the damaged kidney. HGF is a pleiotropic growth factor that plays a vital role in the repair of renal tubular injury [16, 17]. Activation of the Wnt/β-catenin signaling pathway in renal interstitial fibroblasts inhibits downstream HGF/c-met signaling, thereby inhibiting the repair of renal tubules damage by IRI [18]. However, the mechanism of Wnt/β-catenin signaling pathway activation in renal interstitial fibroblasts during AKI is still unclear.

One potential activator is S100A16, a newly identified member of the calmodulin S100 family. S100A16 is a small molecule acidic Ca^2+^-binding protein that has received attention because of its unique characteristics that differ from those of other members of the S100 family [19, 20]. Previous studies have shown that the members of the S100 protein family are regarded as multi-function signal factors involved in various processes of intracellular or extracellular regulation. Functionally, the S100 protein family can regulate cell proliferation, differentiation, migration, apoptosis, and autoimmunity [21, 22]. S100A16, in particular, is widely expressed in human tissues and is a highly conserved protein in mammals [23, 24]. It is revealed as a novel lipogenic factor that promotes the formation of lipid droplets during the differentiation of 3T3-L1 cells. S100A16 also negatively regulates insulin sensitivity in 3T3-L1 cells. These results have suggested that S100A16 is closely associated with obesity [25]. In our previous study, we have reported that the protein expression of S100A16 is significantly increased in the kidneys of unilateral ureteral occlusion (UUO) mice and the characteristic pathological changes of renal tubulointerstitial fibrosis appeared in the kidney of S100A16 transgenic mice, indicating a positive relationship between S100A16 and tubulointerstitial fibrosis. Moreover, S100A16 is also highly expressed in kidney biopsy specimens from patients with various clinical nephropathy [26]. However, the role of S100A16 in AKI remains unclear. Some studies reported that S100 protein can increase Wnt/β-catenin signaling [27, 28]. Therefore, it should be significant to study the effects of S100A16 on AKI associated with Wnt/β-catenin signaling.

In the present study, we investigated the biological function and molecular mechanism of S100A16 in AKI using a S100A16 knockout mouse model and renal interstitial fibroblasts (NRK-49F cells).

## Results

### S100A16 knockout attenuates kidney injury in mouse AKI model

**Fig. 1:**
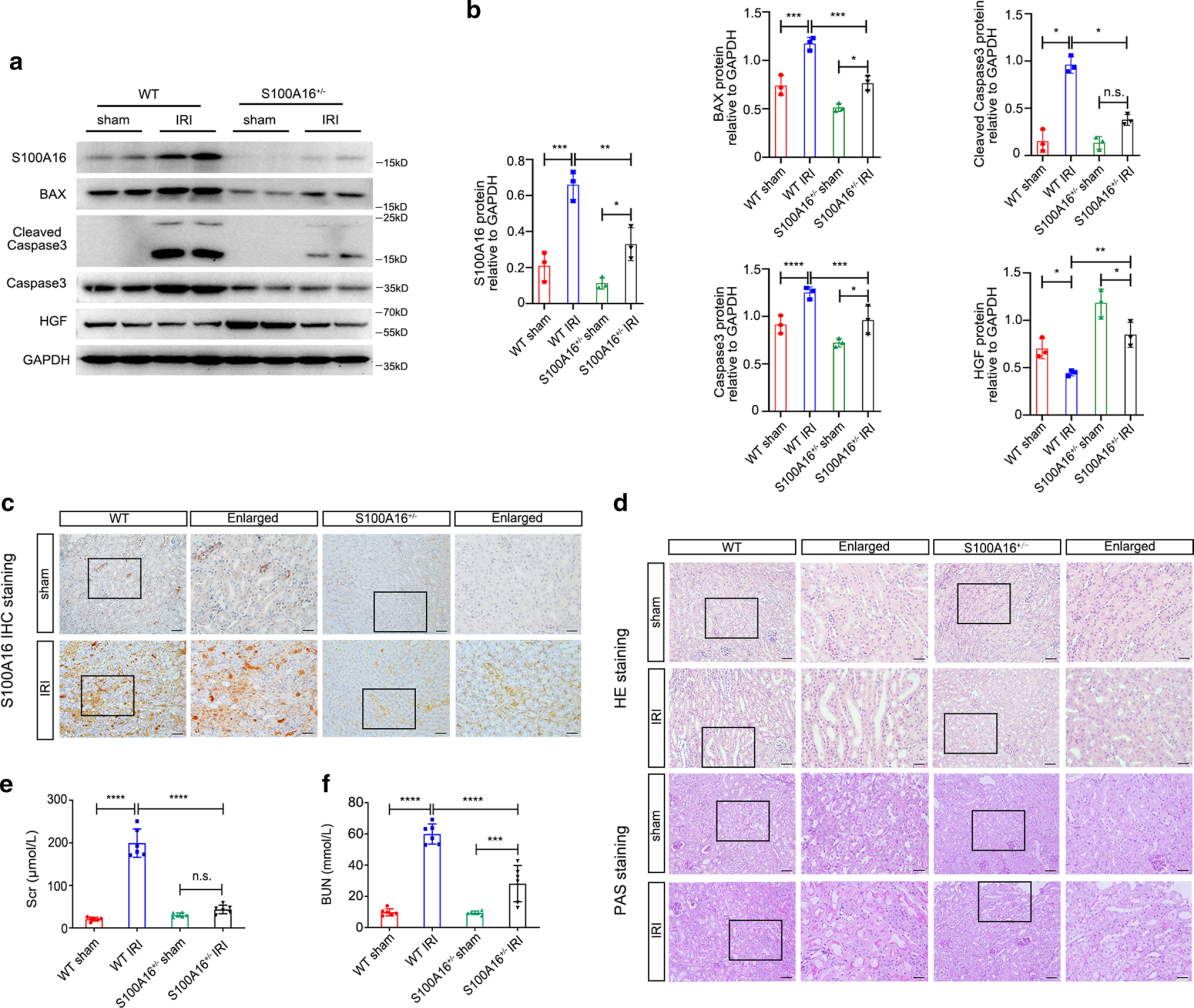
Knockout of S100A16 in mice reduces kidney injury after AKI. **a** The protein expressions of S100A16, BAX, Cleaved Caspase3, Caspase3 and HGF were tested by western blotting using kidney tissues from WT mice and S100A16^+/−^ mice at 1 day after IRI and compared with tissues from sham mice. **b** Quantitation of immunoblot data for S100A16, BAX, Cleaved Caspase3, Caspase3 and HGF as in **a**. ****P < 0.0001, ***P < 0.001, **P < 0.01, *P < 0.05, *n.s.* not significant. *n* = 3; each point represents the expression in a sample pooled from two mice. **c** Representative micrographs in the corticomedullary junction of mice kidneys showed the expression of S100A16 in WT mice and S100A16^+/−^ mice at 1 day after IRI as determined by immunohistochemical staining. Scale bar, 50 μm, 20 μm (Enlarged). **d** Representative micrographs showed morphologic injury in HE and PAS staining sections from the WT mice and S100A16^+/−^ mice at 1 day after IRI. Scale bar, 50 μm, 20 μm (Enlarged). **e** Scr level in the WT mice and S100A16^+/−^ mice at 1 day after IRI, compared with sham mice. ****P < 0.0001, *n.s.* not significant. *n* = 6. **f** BUN in the WT mice and S100A16^+/−^ mice at 1 day after IRI, compared with sham mice. ****P < 0.0001, ***P < 0.001. *n* = 6

We investigated the pathogenic relevance of S100A16 in AKI by first examining the S100A16 protein expression in the kidney tissues in WT mice and S100A16^+/−^ mice with or without an IRI procedure. Of note, S100A16 homozygous knockout is embryonic lethal. As shown in Fig. 1a, S100A16 expression was strongly increased in the kidneys of IRI WT mice than in the WT sham group. Interestingly, although of the same direction, IRI had less effect on S100A16 expression in S100A16^+/−^ groups. The quantified data are shown in Fig. 1b. Further assessment of S100A16 expression by immunohistochemistry staining (IHC) in kidney samples revealed a strong upregulation of S100A16 expression in the renal interstitium of WT IRI mice, but only a mild change in kidneys of the S100A16^+/−^ IRI animals (Fig. 1c). These results were consistent with the biochemistry data.

We then examined the histological changes in WT and S100A16^+/−^ mouse kidneys with or without the IRI procedure. Renal tissues stained with hematoxylin–eosin (HE) staining showed tubular dilatation in WT mice after IRI, while periodic acid-Schiff (PAS) staining showed expansion of the inner edges of the renal tubules, rupture of the renal tubular basement membranes, and appearance of protein casts and cell debris in the lumen after renal IRI. By contrast, the S100A16^+/−^ mice showed a marked reduction in the pathology of renal injury induced by IRI (Fig. 1d). The tubular injury score was significantly decreased in S100A16^+/−^ mice compared with WT mice after IRI induction (Fig. S1a). As shown in Fig. 1e, f, tests of serum creatinine (Scr) and blood urea nitrogen (BUN) conducted 24 h after IRI showed significant elevation in WT mice after IRI compared to the sham group. However, the S100A16^+/−^ IRI mice had markedly lower levels of Scr and BUN compared with WT IRI mice. Western blot analyses also revealed that S100A16 heterozygous knockout effectively inhibited the IRI-induced upregulation of pro-apoptotic genes including BAX, Cleaved Caspase3, and Caspase3, and reversed the destruction of HGF, the renal repair cytokine (Fig. 1a, b). These data indicated that S100A16 knockout has a protective role in renal IRI, likely through attenuating the degree of renal ischemia and hypoxia injury.

### S100A16 knockout reduces activation of Wnt/β-catenin pathway in AKI mice

**Fig. 2:**
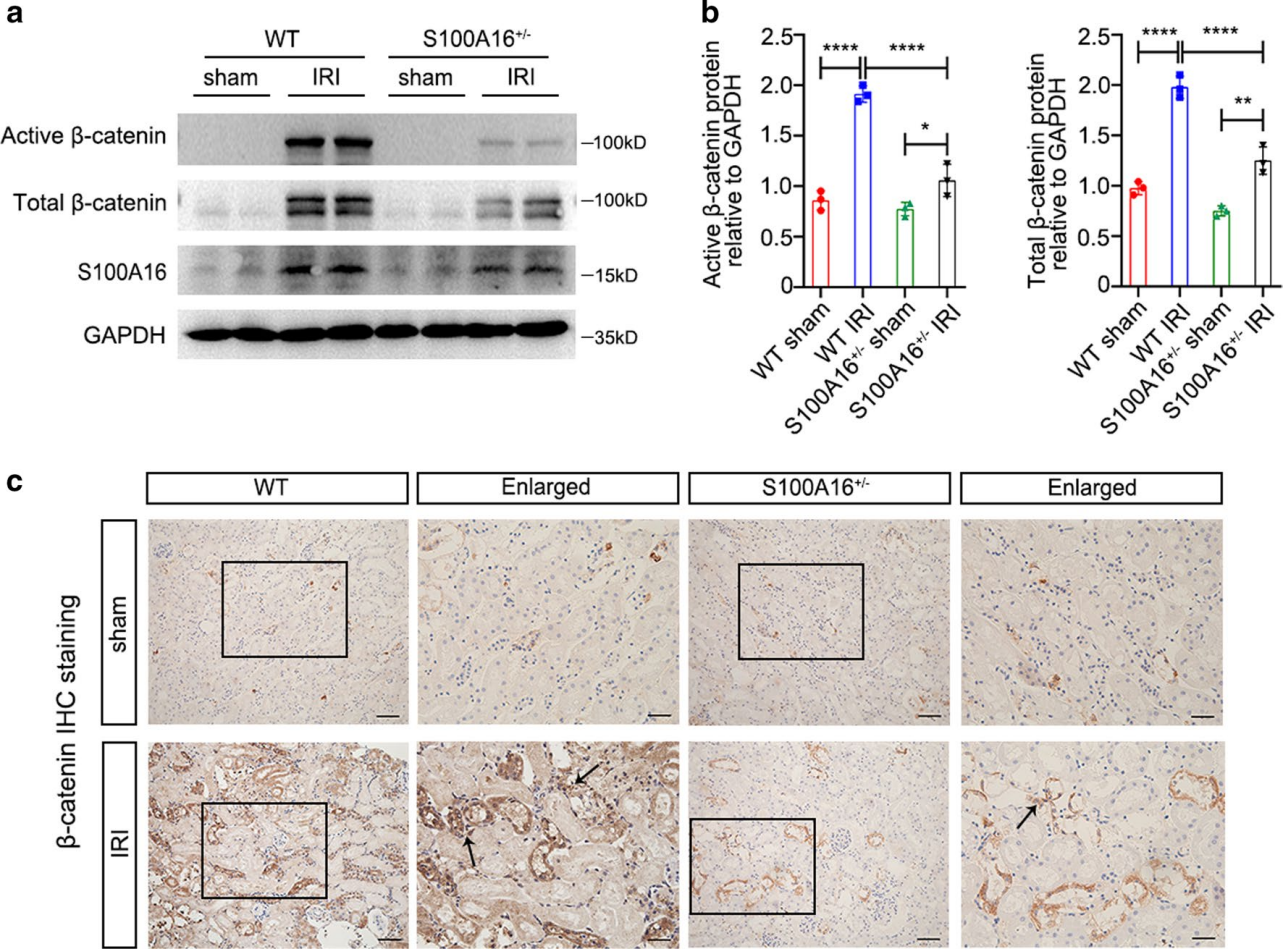
S100A16 knockout in mice disrupts Wnt/β-catenin pathway activation after AKI. **a** The expressions of Active β-catenin, Total β-catenin, and S100A16 were detected by western blotting using kidney tissues from WT mice and S100A16^+/−^ mice at 1 day after IRI, and sham mice were used as control. **b** Quantitation of western blot data for Active β-catenin and Total β-catenin proteins as in **a**. ****P < 0.0001, **P < 0.01, *P < 0.05. *n* = 3; each point represents the expression in a sample pooled from two mice. **c** Representative micrographs in the corticomedullary junction of mice kidneys showed expression of β-catenin in WT mice and S100A16^+/−^ mice at 1 day after IRI as determined by immunohistochemical staining. Black arrows point to the β-catenin IHC positive staining in the renal interstitium. Scale bar, 50 μm, 20 μm (Enlarged)

HGF is a downstream factor of Wnt/β-catenin signaling pathway. S100A16 knockout increased HGF expression as shown in Fig. 1a. Therefore, we further explored whether S100A16 is involved in the activation of the Wnt/β-catenin signaling pathway induced by renal IRI. As shown in Fig. 2a, the expressions of Active β-catenin (non-phospho β-catenin at Ser33/37/Thr41) and Total β-catenin were significantly upregulated in WT mice at 24 h after the IRI procedure compared to the corresponding sham groups. This finding indicated that the Wnt/β-catenin signaling pathway is activated by IRI in this mouse model. However, the expressions of β-catenin-driven genes after IRI were significantly lower in the S100A16^+/−^ kidneys than in WT injured kidneys. Together, these data indicated that S100A16 knockout diminished the IRI-induced activation of Wnt/β-catenin pathway. The quantified data for Active β-catenin and Total β-catenin expression are shown in Fig. 2b.

We then interrogated the relationship between Wnt signals and S100A16. Real-time PCR analyses showed a sustained activation of numerous Wnt ligands, including Wnt2, Wnt3, Wnt4, Wnt7a, Wnt7b, Wnt8a, Wnt10b, and Wnt16, at 24 h after the IRI treatment in WT mice compared to WT sham mice (Fig. S1b–i). By contrast, the mRNA levels of Wnt ligands relative to β-actin were close to the sham WT baseline in the S100A16^+/−^ IRI mice (Fig. S1b–i). These results confirmed that S100A16 positively regulates the activation of the Wnt/β-catenin signaling pathway in the kidney after IRI. The IHC staining data of β-catenin confirmed Wnt/β-catenin activation after IRI in WT mice both in renal tubules and interstitium; β-catenin immunoreactivity was more modestly increased after IRI in S100A16^+/−^ mice and was more sparsely distributed compared to WT mice (Fig. 2c).

### S100A16 directly participates in hypoxia/reoxygenation-induced Wnt/β-catenin signaling activation in renal fibroblasts

**Fig. 3:**
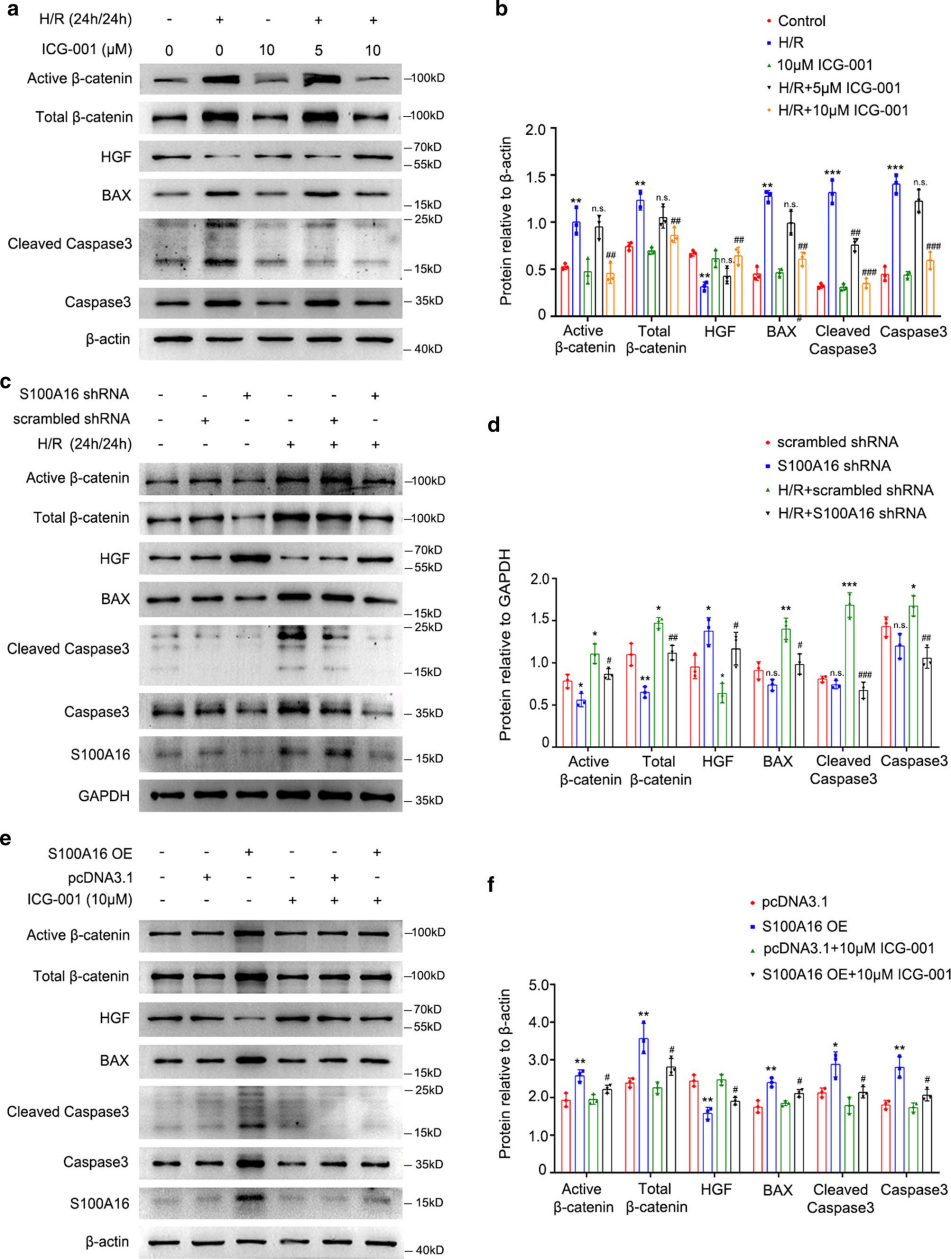
Wnt/β-catenin pathway activation is affected by regulating S100A16 in hypoxia/reoxygenation-induced NRK-49F cells. **a** Western blot analyses showed that ICG-001 blocked Wnt/β-catenin pathway activation induced by H/R in NRK-49F cells. Cell lysates after ICG-001 treatment as indicated were immunoblotted with antibodies against Active β-catenin, Total β-catenin, HGF, BAX, Cleaved Caspase3, Caspase3 and β-actin. **b** Quantitation of western blot data for Active β-catenin, Total β-catenin, HGF, BAX, Cleaved Caspase3 and Caspase3 proteins as in **a**. ***P < 0.001, **P < 0.01, versus control; ^###^*P* < 0.001, ^##^*P* < 0.01, *n.s.* not significant, versus H/R alone. *n* = 3. **c** Western blot analyses showed that knockdown S100A16 inhibited H/R-induced the increased expressions of Active β-catenin, Total β-catenin, BAX, Cleaved Caspase3, and Caspase3 in NRK-49F cells. Knockdown S100A16 also recovered the HGF expression. **d** Quantitation of western blot data for Active β-catenin, Total β-catenin, HGF, BAX, Cleaved Caspase3, and Caspase3 proteins as in **c**. ***P < 0.001, **P < 0.01, *P < 0.05, *n.s.* not significant, versus scrambled shRNA; ^###^*P* < 0.001, ^##^*P* < 0.01, ^#^*P* < 0.05, versus H/R + scrambled shRNA. *n* = 3. **e** Western blot analyses displayed the elevated protein expressions of Active β-catenin, Total β-catenin, BAX, Cleaved Caspase3, and Caspase3 in S100A16 overexpressing NRK-49F cells, and these genes could be impeded by ICG-001. By contrast, the decreased HGF expression caused by S100A16 overexpression could be recovered by ICG-001. **f** Quantified Active β-catenin, Total β-catenin, HGF, BAX, Cleaved Caspase3, and Caspase3 protein levels in **e**. **P < 0.01, *P < 0.05, versus pcDNA3.1; ^#^*P* < 0.05, versus S100A16 OE. *n* = 3

To identify the localization of S100A16 in vivo, we performed the double immunofluorescence staining for S100A16 and Platelet-derived growth factor receptor beta (PDGFRβ) in AKI kidney tissues. The images revealed that S100A16 was highly expressed in PDGFRβ positive renal fibroblasts (Fig. S2). To further investigate the role of S100A16 in AKI linked to Wnt/β-catenin signaling, rat renal interstitial fibroblasts (NRK-49F cells) were cultured under basal or hypoxic conditions, with or without different concentrations (0, 5, and 10 μM) of ICG-001, a Wnt/β-catenin signaling pathway inhibitor. Cell lysates from NRK-49F cells were tested by western blotting for protein expressions of β-catenin-driven genes and cell injury-related genes. As shown in Fig. 3a, exposure of NRK-49F cells to hypoxia/reoxygenation (H/R) triggered increased expressions of Active β-catenin and Total β-catenin, and markedly evoked the expressions of BAX, Cleaved Caspase3 and Caspase3. Treatment with 10 μM ICG-001 significantly diminished this H/R-induced upregulation of β-catenin-driven genes and pro-apoptotic genes in NRK-49F cells. In contrast, the protein expression of HGF, a downstream target of Wnt/β-catenin pathway, was restored by ICG-001 treatment in NRK-49F cells under H/R condition. These results indicated that injury induced in renal interstitial fibroblasts by H/R facilitates the activation of Wnt/β-catenin signaling pathway and inhibits downstream HGF expression. Quantitative results are shown in Fig. 3b.

We then used S100A16 knockdown and S100A16 overexpressing in NRK-49F cells to further investigate the role of S100A16 in renal hypoxia injury. We knocked down S100A16 expression in NRK-49F cells using shRNA and used scrambled shRNA as control. As shown in Fig. 3c, knockdown of S100A16 in NRK-49 cells reduced the protein expressions of Active β-catenin and Total β-catenin induced by H/R. Western blots also revealed the increased protein expressions of apoptosis-related genes and the decreased protein expression of HGF caused by H/R were effectively reversed by S100A16 knockdown. Quantitative results are shown in Fig. 3d.

Overexpression of S100A16, on the other hand, upregulated the protein expressions of Active β-catenin and Total β-catenin along with apoptosis-related genes, and this upregulation was inhibited by ICG-001. Notably, the protein expression of HGF was markedly reduced by S100A16 overexpression, which is also reversed by the use of 10 μM ICG-001 (Fig. 3e). The quantitative results are shown in Fig. 3f.

### S100A16 regulates HRD1 during Wnt/β-catenin pathway activation

In our previous study, we have identified that CK1α, a β-catenin degraded complex member, is a substrate for HRD1, an E3 ubiquitin ligase [29]. In the present study, we examined whether HRD1 is associated with S100A16 in the process of Wnt/β-catenin pathway activation.

**Fig. 4:**
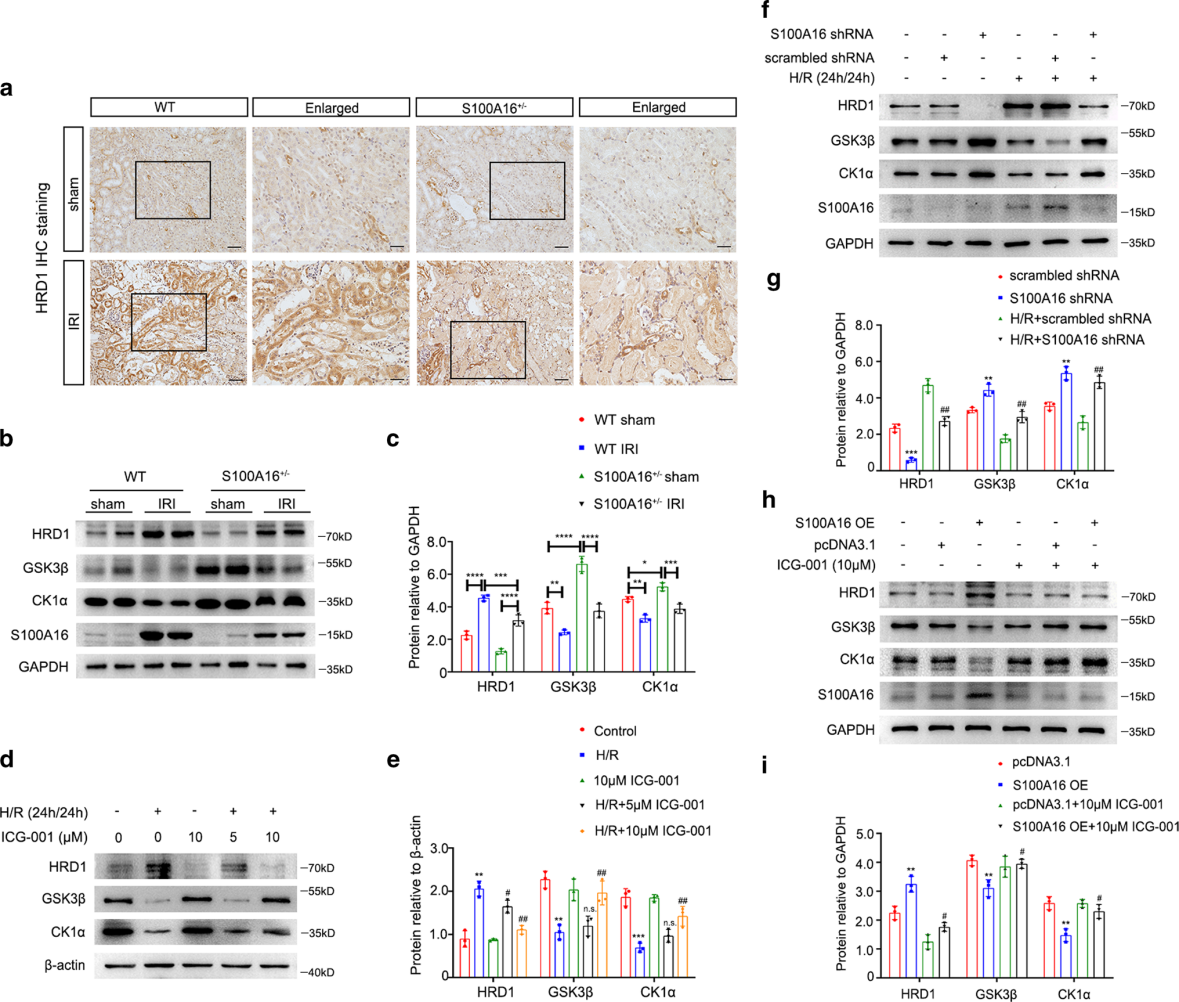
HRD1 involves in the activation of Wnt/β-catenin signaling pathway and is regulated by S100A16 in injured renal fibroblasts. **a** Representative micrographs in the corticomedullary junction of mice kidneys showed the expression of HRD1 in WT mice and S100A16^+/−^ mice at 1 day after IRI, as determined by immunohistochemical staining. Scale bar, 50 μm, 20 μm (Enlarged). **b** HRD1, GSK3β and CK1α expressions were tested using kidney tissues from WT mice and S100A16^+/−^ mice at 1 day after IRI compared with sham mice by western blot assays. **c** Quantitation of immunoblot data for HRD1, GSK3β and CK1α proteins as in **b**. ****P < 0.0001, ***P < 0.001, **P < 0.01, *P < 0.05. *n* = 3; each point represents the expression in a sample pooled from two mice. **d** Western blot analyses showed that ICG-001 blocked the increased HRD1 expression induced by H/R in NRK-49F cells, and ICG-001 also recovered the expressions of GSK3β and CK1α. Cell lysates after various treatments, as indicated, were immunoblotted with antibodies against HRD1, GSK3β, CK1α and β-actin. **e** Quantitation of western blot data for HRD1, GSK3β and CK1α proteins as in **d**. ***P < 0.001, **P < 0.01, versus control; ^##^*P* < 0.01, ^#^*P* < 0.05, n.s. not significant, versus H/R alone. *n* = 3. **f** Western blot analyses showed that knockdown of S100A16 inhibited the HRD1 expression and enhanced the expressions of GSK3β and CK1α in normal or hypoxia condition. **g** Quantitation of western blot data for HRD1, GSK3β and CK1α proteins as in **f**. ***P < 0.001, **P < 0.01, versus scrambled shRNA; ^##^*P* < 0.01, versus H/R + scrambled shRNA. *n* = 3. **h** Western blots showed that overexpression of S100A16 increased the HRD1 expression, but it was impeded by ICG-001 in NRK-49F cells. The GSK3β and CK1α expressions were opposite to HRD1. **i** Quantified HRD1, GSK3β and CK1α protein levels in **h**. **P < 0.01, versus pcDNA3.1; ^#^*P* < 0.05, versus S100A16 OE. *n* = 3

IHC staining showed a marked increase in the expression of HRD1 in the WT kidney tissues following IRI, but the upregulation of HRD1 in S100A16^+/−^ kidney was not as larger as in WT ones (Fig. 4a). These differences in HRD1 expression were confirmed by western blot (Fig. 4b), and by real-time PCR (Fig. S3a). The GSK3β and CK1α in the kidney lysates were lower after IRI than those before IRI in WT groups, whereas in the S100A16^+/−^ IRI kidney lysates, the protein expressions of GSK3β and CK1α were restored to 95% compared with WT IRI kidneys (Fig. 4b). Quantitative results of the expressions of HRD1, GSK3β and CK1α are shown in Fig. 4c. These results indicated that IRI induces increased HRD1 protein expression and decreased protein expressions of GSK3β and CK1α in mice, and S100A16 knockout can reduce these changes.

We then explored the potential involvement of HRD1 in the activation of Wnt/β-catenin pathway during renal ischemia and hypoxia, by pretreating NRK-49F cells under the H/R condition with different regulation of S100A16. We found that HRD1 expression was increased under hypoxic stimulation and was attenuated by ICG-001 (Fig. 4d, e). H/R also increased the mRNA level of HRD1, as detected by real-time PCR (Fig. S3b). We then transfected S100A16 shRNA into NRK-49F cells to knock down S100A16 expression. HRD1 expression was decreased in NRK-49F cells transfected with S100A16 shRNA compared to the normal control or scrambled shRNA control, and S100A16 knockdown also impeded the H/R-induced upregulation of HRD1. By contrast, the changes in the protein expression levels of GSK3β and CK1α were opposite to those of HRD1 (Fig. 4f, g). We also observed that the protein expression of HRD1 was augmented when S100A16 was overexpressed in NRK-49F cells, and its augmentation was reversed by treatment with 10 μM ICG-001. The protein expression changes in GSK3β and CK1α were opposite to those of HRD1 (Fig. 4h, i). These data suggested that S100A16 potentiates the activation of Wnt/β-catenin signaling pathway by upregulating HRD1.

### HRD1 physically binds and promotes the ubiquitination and degradation of both GSK3β and CK1α

**Fig. 5:**
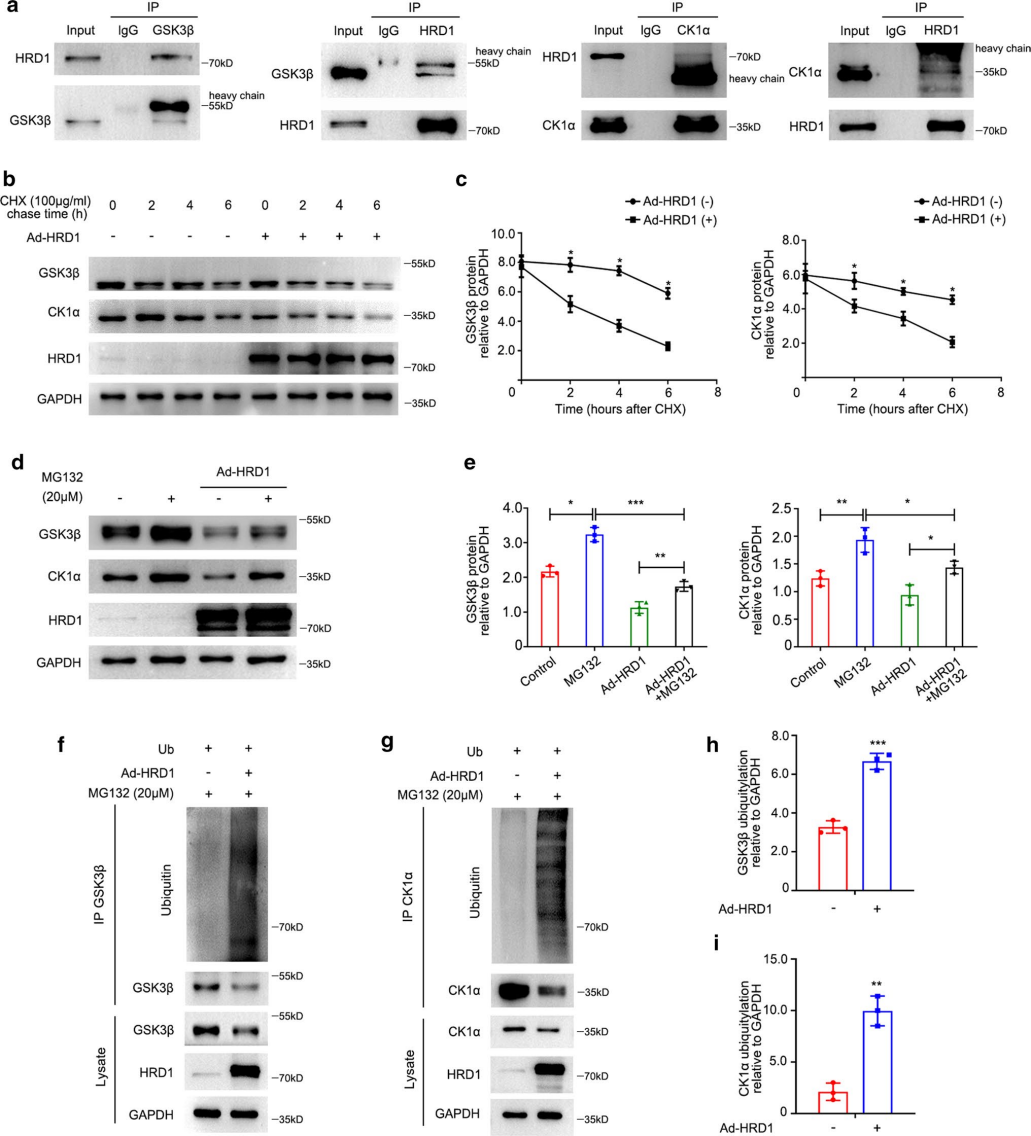
HRD1 degrades both GSK3β and CK1α via the ubiquitin–proteasome pathway in NRK-49F cells. **a** The interaction between HRD1 and either GSK3β or CK1α was detected in the co-IP analysis in NRK-49F cells. **b** A cycloheximide chase was performed to establish the time course of GSK3β or CK1α biogenesis. NRK-49F cells were infected with or without Ad-HRD1 for 48 h, and then the cells were treated with CHX (100 μg/ml) for 0, 2,4 or 6 h. The expressions of GSK3β and CK1α in whole-cell lysates were measured by western blotting. **c** Quantitation of western blot data for GSK3β and CK1α proteins as in **b**. *P < 0.05. *n* = 3. **d** The presence of MG132 (20 µM) increased the expressions of GSK3β and CK1α with or without Ad-HRD1 infection compared with controls in NRK-49F cells. **e** Quantification of GSK3β and CK1α protein expressions from experiments as shown in **d**. ***P < 0.001, **P < 0.01, *P < 0.05. *n* = 3. **f** More ubiquitin conjugated to GSK3β was detected in the cells overexpressing HRD1 compared with no HRD1 transfection. **g** More ubiquitin conjugated to CK1α was detected in the cells overexpressing HRD1 compared with no HRD1 transfection. **h** Quantification of ubiquitin conjugated to GSK3β as in **f**, normalized to GAPDH expression. ***P < 0.001. *n* = 3. **i** Quantification of ubiquitin conjugated to CK1α as in **g**, normalized to GAPDH expression. **P < 0.01. *n* = 3

We then conducted co-immunoprecipitation (co-IP) experiments to examine the interaction between HRD1 and GSK3β, as well as the interaction between HRD1 and CK1α, in NRK-49F cells using antibodies against GSK3β or CK1α, to isolate proteins from NRK-49F cells. The isolated proteins were then examined by western blotting to detect binding signals of HRD1. IgG was used as a control which yielded no HRD1 positive signal in immunoprecipitations (IPs). The binding signals of GSK3β or CK1α with HRD1 were detected by anti-HRD1 antibody in IPs, and subsequently blotting with GSK3β or CK1α antibody. As shown in Fig. 5a, we detected physical HRD1 bindings with GSK3β or CK1α in NRK-49F cell lysates. Double immunofluorescence staining confirmed that HRD1 colocalized with GSK3β or CK1α in the cytoplasm of NRK-49F cells (Fig. S4a, b), in agreement with the co-IP analysis.

We also explored the effect of HRD1 on the stability of GSK3β or CK1α expression by performing a cycloheximide (CHX, a protein translation inhibitor) chase assay. Adenovirus HRD1 (Ad-HRD1) was transfected into NRK-49F cells. There was around a 20-fold increase in the expression of HRD1 in NRK-49F cells with transfection of Ad-HRD1 compared to that in non-transfection of Ad-HRD1 (Fig. 5d). Then, the cells were treated with CHX after 48 h Ad-HRD1 transfection, for 0, 2, 4 or 6 h to block the synthesis of new polypeptides. As shown in Fig. 5b, c, the rate of protein disappearance of GSK3β or CK1α was more rapid in the Ad-HRD1 transfected cells than in the control group, indicating that HRD1 is involved in promoting protein degradation of both GSK3β and CK1α. Therefore, overexpressed HRD1 in NRK-49F cells results in shorter half-lives for both GSK3β and CK1α protein expressions.

As shown in Fig. 5d, e, treatment with MG132, a proteasome inhibitor, increased the protein expressions of both endogenous GSK3β and endogenous CK1α compared to each corresponding control group, but Ad-HRD1 did not affect mRNA level of either GSK3β or CK1α (Fig. S5a and S5b). These results implied that HRD1 degrades both GSK3β and CK1α via the ubiquitin–proteasome degradation pathway. We verified this speculation by transfecting ubiquitin (Ub) plasmids into NRK-49F cells with or without Ad-HRD1 infection. GSK3β or CK1α was immunoprecipitated and the protein expression of ubiquitin was observed by western blotting. We detected a higher protein expression of ubiquitin in the cells infected with Ad-HRD1 than in the cells without Ad-HRD1 infection (Fig. 5f–i). These findings verified that HRD1 promotes the ubiquitination of both GSK3β and CK1α in NRK-49F cells.

### S100A16 participates in AKI by mediating the downregulation of both GSK3β and CK1α through HRD1 in renal fibroblasts

**Fig. 6:**
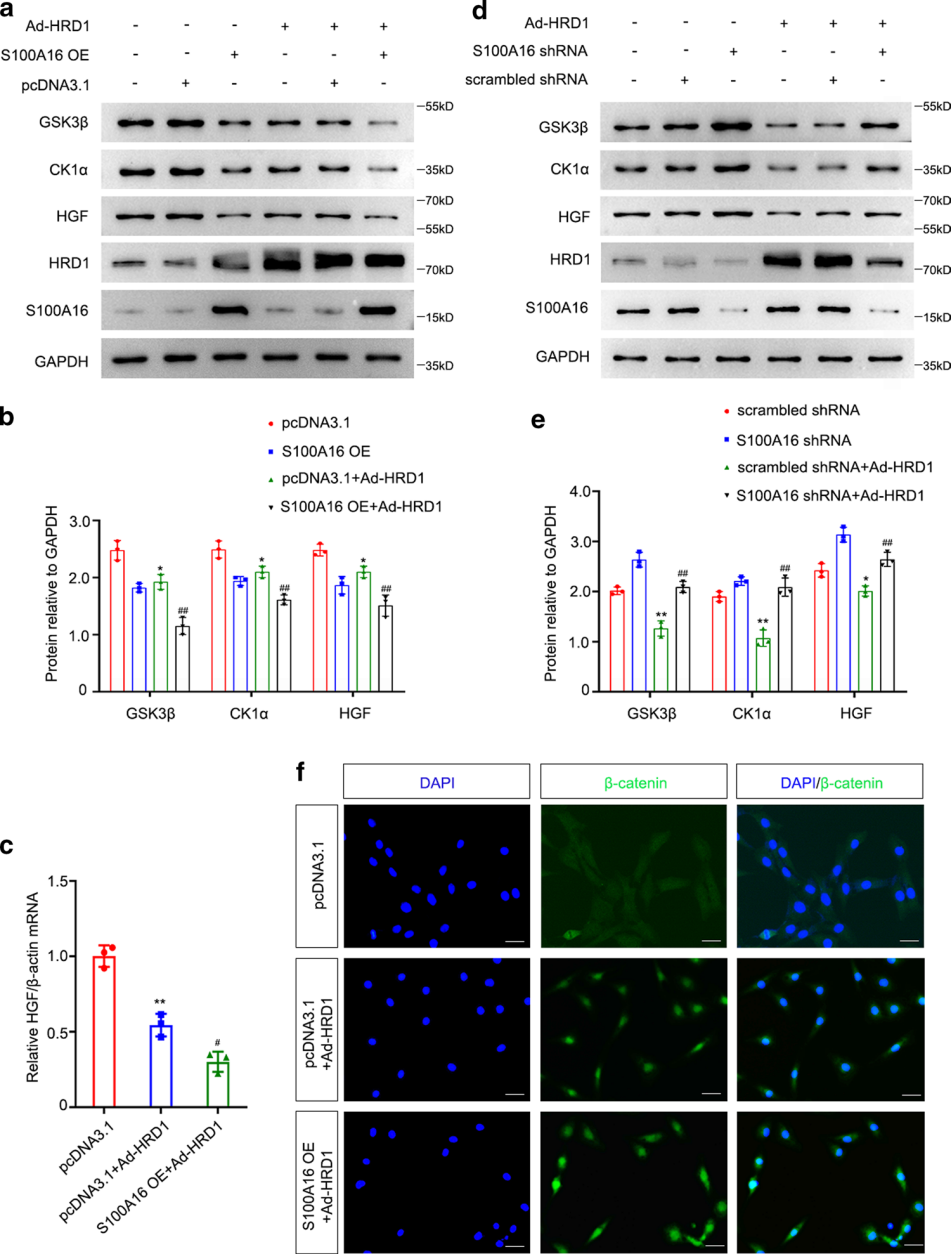
S100A16 down-regulates the expressions of both GSK3β and CK1α by HRD1 to affect downstream HGF in NRK-49F cells. **a** Western blot analyses showed that overexpression of S100A16 aggravated the downregulated expressions of GSK3β, CK1α, and HGF induced by Ad-HRD1 in NRK-49F cells. **b** Quantitation of western blot data for GSK3β, CK1α, and HGF proteins, as in **a**. *P < 0.05, versus pcDNA3.1; ^##^*P* < 0.01, versus pcDNA3.1 + Ad-HRD1. *n* = 3. **c** Real-time qPCR demonstrated that overexpression of S100A16 aggravated the reduction of relative HGF/β-actin mRNA level induced by Ad-HRD1 compared with the Ad-HRD1 treatment alone in NRK-49F cells. **P < 0.01, versus pcDNA3.1; ^#^*P* < 0.05, versus pcDNA3.1 + Ad-HRD1. *n* = 3. **d** Western blot analyses showed that knockdown S100A16 recovered Ad-HRD1-induced the down-regulated expressions of GSK3β, CK1α, and HGF. **e** Quantitation of western blot data for GSK3β, CK1α and HGF proteins, as in **d**. **P < 0.01, *P < 0.05, versus scrambled shRNA; ^##^*P* < 0.01, versus scrambled shRNA + Ad-HRD1. *n* = 3. **f** Representative images showed the accumulation of β-catenin fluorescence in the nucleus of HRD1-overexpressing NRK-49F cells. The β-catenin fluorescence was amplified after overexpressing S100A16 in the HRD1-overexpressing NRK-49F cells. Scale bar, 20 μm

We explored the possible molecular mechanism of S100A16 in AKI via HRD1-mediated Wnt/β-catenin pathway activation by co-transfecting Ad-HRD1 and S100A16 overexpressing (OE) plasmids into NRK-49F cells to detect the protein expressions of GSK3β, CK1α, and HGF. These results showed markedly decreased protein expression levels of GSK3β, CK1α and HGF in NRK-49F cells co-transfected with Ad-HRD1 and S100A16 OE plasmids compared to control groups (Fig. 6a, b). The mRNA levels of HGF, determined by real-time PCR, were consistent with protein expression values, as shown in Fig. 6c. The degradation of both GSK3β and CK1α by Ad-HRD1 in NRK-49F cells was suppressed by transfection with S100A16 shRNA compared with cells transfected scrambled shRNA. The S100A16 shRNA transfection restored the HRD1-mediated downstream HGF suppression, as shown in Fig. 6d, e. These data demonstrated that S100A16 participates in AKI by promoting HRD1-mediated degradation of both GSK3β and CK1α in NRK-49F cells.

The immunofluorescence staining of β-catenin confirmed that β-catenin was mainly expressed in the cytoplasm of NRK-49F cells transfected with vector only, but positive signals of β-catenin were evident in the nucleus of NRK-49F cells infected with Ad-HRD1, and these signals were even stronger in the nucleus of cells transfected with the Ad-HRD1 and S100A16 OE combination (Fig. 6f).

## Discussion

The pathogenesis of AKI is a complex process. AKI is a complication with high clinical morbidity and mortality, often caused by specific factors, such as hypotension, bacterial infection, sepsis, and toxic invasion, that lead to microcirculation damage, inflammation, and renal tubular injury [30]. IRI is the main culprit in AKI. The oxygen supply to the kidney is extremely rich, so the kidney is one of the organs most vulnerable to oxygen-free radical damage in the body and is highly sensitive to IRI [31]. The pathophysiological process of IRI is complicated and involves energy depletion due to hypoxia, inflammatory response, oxidative stress and apoptosis. The prevention and treatment of IRI are, therefore, of great significance to AKI patients [32]. Wnt/β-catenin signaling has been known to be a key pathway for normal kidney organogenesis and the development of kidney disease. Both tubular cells and interstitial fibroblasts in the kidney response to Wnt/β-catenin signaling, and both cell types can express Wnt proteins[33]. In AKI, previous studies have suggested that the role of the Wnt/β-catenin signaling pathway is a double-edged sword: activation of the transient Wnt/β-catenin signaling pathway causes self-repair of damaged tubular epithelial cells, but sustained Wnt/β-catenin signaling pathway activation results in activation of the renin-angiotensin system (RAS), inflammation, and excessive deposition of extracellular matrix (ECM) [34, 35]. However, a recent report has proposed a new view whereby activation of Wnt/β-catenin signaling pathway induced by short-term IRI in fibroblasts plays a potential role in AKI. In this study, the deletion of β-catenin in fibroblasts increased the expression of HGF in the kidney and reduced the IRI-induced renal damage [18]. HGF secreted by fibroblasts promotes the survival and proliferation of tubule cells and disrupts NF-κB signaling to inhibit inflammation. Thus, HGF plays an important role in the kidney self-repair after AKI [36, 37]. These findings promoted us to explore the related mechanism of Wnt/β-catenin signaling pathway in fibroblasts in AKI and the potential role of S100A16.

S100A16 belongs to the calcium-binding protein S100 family and is a newly discovered member that is expressed in a variety of tissues. The S100 family is widely expressed in human tissues and contains proteins characterized by two EF helix structures with different calcium affinities. The C-terminal EF helix structure consists of a typical Ca^2+^-binding loop formed by 12 amino acids, while the N-terminal EF helix structure contains 14 S100 protein-specific amino acids [38]. The S100 protein is involved in a range of biological processes, and S100 protein is also associated with multiple diseases, such as inflammation, neurodegenerative diseases, depression, Down's syndrome, cystic fibrosis, and cancers [39]. Multiple members of the S100 family are markers of certain tumors, such as S100A7, S100A13, S100A14 [19, 20]. Notably, according to a recent report, S100A8/A9 could cause cardiomyocytes death in response to ischemic/reperfusion injury [40]. Therefore, we speculated that the expressions of S100 family members may be associated with ischemia injury.

Other reports have indicated that S100 protein can increase Wnt signaling; for example, the role of S100A8 is partially dependent on the activation of Wnt/β-catenin signaling, and inhibition of S100A4 expression impedes colon cancer progression caused by the Wnt/β-catenin signaling pathway [41, 42]. For these reasons, we anticipated that members of the S100 family would be involved in the activation of Wnt/β-catenin signaling. S100A16 is specific in its molecular structure, suggesting that it may have a more complex regulatory mechanism. S100A16 has an EF-helix structure bound to Ca^2+^ at the C-terminus and N-terminus, whereas the C-terminus is identical to the traditional EF-helix, consisting of 12 highly conserved amino acids. The N-terminal includes the loop consisting of 15 amino acids and it lacks the glutamic acid residue at the last position that normally facilitates the coordination of the calcium-binding site. The binding of Ca^2+^ by S100A16 exposes the hydrophobic binding site to allow binding of the target protein and inductions of various biological functions [19, 43]. Few reports have been published on the function of S100A16 gene and most studies have focused on its relationship with tumors, cell proliferation and tissue metabolism.

Previous studies have reported high expression of S100A16 in malignant tumors and the promotion of cell invasion and tumor development. In addition, S100A16 is now viewed as a novel adipogenic factor involved in glycolipid metabolism [44–46], but its functional role in AKI is largely unknown. Here, we have found that S100A16 is highly expressed in the kidney after 24 h IRI surgery in mice. S100A16 knockout also relieves the degree of renal injury in vivo and reduces Wnt/β-catenin signaling activated by IRI. S100A16 might be a potential clinic-relevant target in renal injury. Based upon single nuclear RNAseq data (http://humphreyslab.com/SingleCell/) done on IRI samples, the gene expression of S100A16 is localized mainly to the macula densa. But our studies here also showed an involvement of the S100A16 in renal interstitial fibroblasts in the positive regulation of activation of hypoxia-induced Wnt/β-catenin signaling pathway. Some studies reported that the activation of fibroblast could promote short-term repair after AKI, on the other hand, it was reported that robust fibroblasts could transform to myofibroblasts with persisting Wnt/β-catenin activity after moderate-severe AKI. Therefore, the functional role of interstitial fibroblast during AKI needs more investigation.

The stability of key molecules, such as β-catenin and its degradation complex, in the Wnt/β-catenin signaling pathway is regulated by the ubiquitin ligase E3 complex, and these ubiquitin ligase complexes are regulated by different signaling molecules in different tissues and cells [47]. The members of β-catenin degradation complex are significant players in the Wnt/β-catenin signaling pathway and are the key molecules that determine the stability and transcriptional activity of β-catenin. In the absence of Wnt signals, the β-catenin degradation complex member CK1α first causes phosphorylation of β-catenin at the Ser45 site. The β-catenin is then further phosphorylated at the Thr41, Ser37, and Ser33 sites by the action of GSK3β, another member of the β-catenin degradation complex. The β-catenin phosphorylated by GSK3β then binds to E3 ubiquitin ligase β-trcp and is ubiquitinated for proteasome degradation [48, 49]. In our previous study, we have used protein liquid chromatography-mass spectrometry/mass spectrometry (LC–MS/MS) to screen out the β-catenin degradation complex member CK1α as the one of the substrate of the HRD1 E3 ubiquitin ligase [29]. The Ubiquitin–proteasome pathway has essentially three levels: a ubiquitin-activating enzyme (E1), a ubiquitin-conjugating enzyme (E2), and a ubiquitin ligase (E3). Of these, the E3 ubiquitin ligase plays a key role in this ubiquitination process as it determines the specific recognition and binding of ubiquitin molecules and protein substrates [50]. HRD1 is a specific E3 ubiquitin ligase located on the endoplasmic reticulum membrane, where it targets misfolded proteins for degradation in cells, therefore, this ligase is closely related to the development of many diseases [51]. However, the relationship between HRD1 and AKI, and the specific HRD1 substrates that are associated with kidney disease, remains unclear. In our experiments, we found that HRD1 interacts directly with GSK3β and CK1α and targets GSK3β and CK1α for degradation by proteasome pathway ubiquitination.

**Fig. 7:**
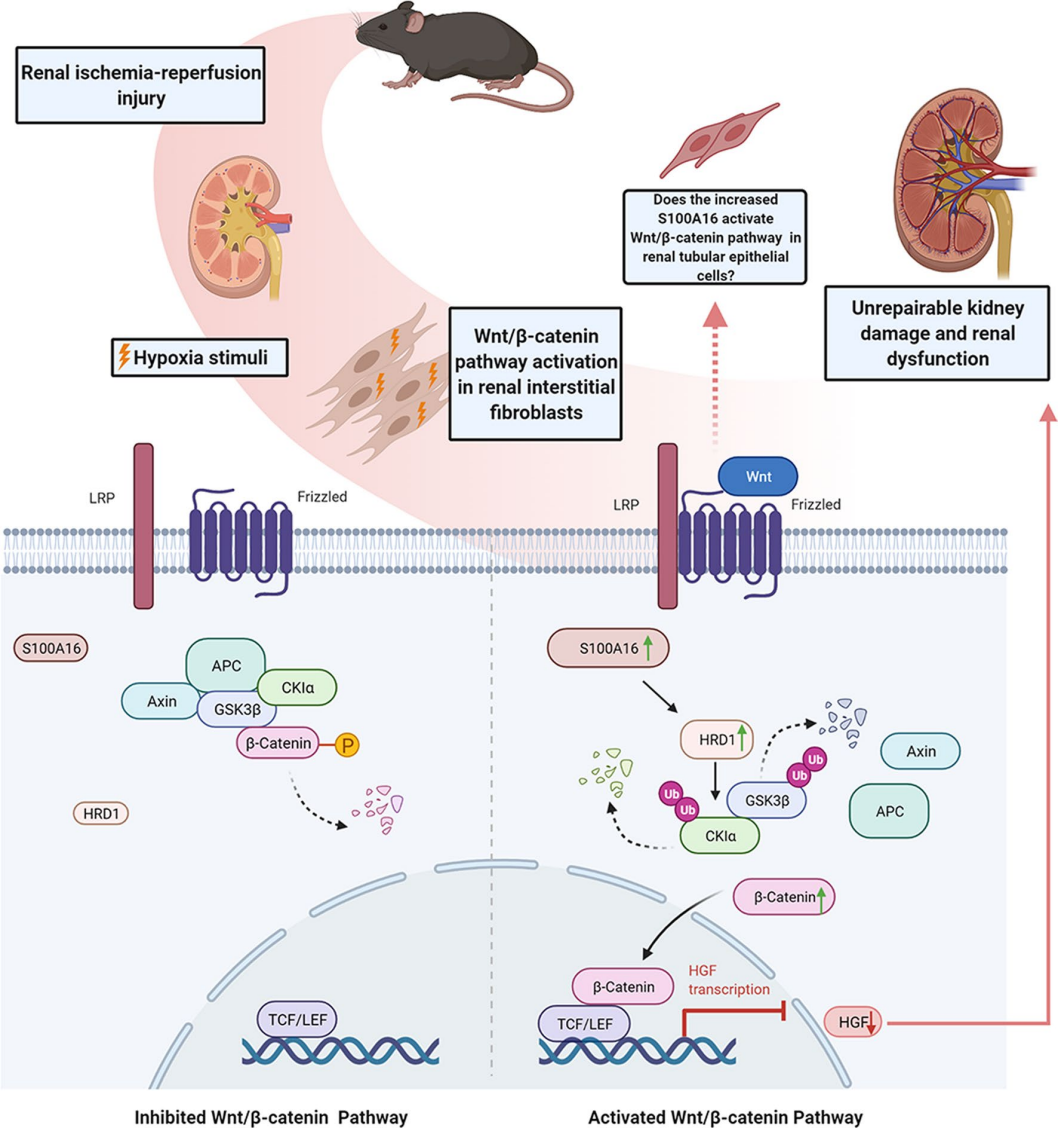
The Wnt/β-catenin signaling activation induced by S100A16–HRD1–GSK3β/CK1α pathway in renal fibroblasts under IRI condition promotes the occurrence of AKI. Schematic diagram shows the increased expression of S100A16 in fibroblasts during renal ischemia and hypoxia. The expression of the E3 ubiquitin ligase HRD1 is elevated, and the members of β-catenin degradation complex, GSK3β and CK1α, are destroyed via the ubiquitin–proteasome pathway, thereby promoting the accumulation of β-catenin and its transfer to the cell nucleus. Activation of the Wnt/β-catenin signaling pathway then inhibits the transcription of the downstream HGF that secreted by fibroblasts, and this procedure eventually leads to unrepairable kidney injury and renal dysfunction

Taken together, the findings in this study have provided insights into how the S100A16 activates Wnt/β-catenin signaling in AKI. We have identified the S100A16–HRD1–GSK3β/CK1α axis as a new Wnt/β-catenin signaling mechanism that is active in renal fibroblasts and potentially other cell types such as tubular epithelial cells under IRI condition. The illustration in Fig. 7 shows our model in which both GSK3β and CK1α, as members of the β-catenin degradation complex, interact with HRD1, an E3 ubiquitin ligase, to target proteins for ubiquitination and degradation in the condition of renal injury; β-catenin is subsequently released and translocated into the nucleus in response to Wnt/β-catenin signaling activation, and the expression of HGF is repressed, leading to severe kidney damage. The results of this study suggest that S100A16 is a novel and promising regulator of the Wnt/β-catenin signaling activation in fibroblasts when AKI occurs.

## Methods and materials

### Mouse ischemia–reperfusion injury models

Heterozygous S100A16 knockout (S100A16^+/−^) mice were acquired from the Model Animal Center of Nanjing University (Contract No. [2009] T67). After breeding heterozygous pairs, heterozygous male mice and their littermate WT male mice (age, 10–12 weeks; body weight, 20–24 g) were used for experiments. All protocols of animal experimentation and maintenance comply with the principles of the Institutional Animal Care and Use Committee of Nanjing Medical University.

IRI model experiments were divided into four groups (*n* = 6 in each group): sham operation group of wild type mice (WT sham), IRI operation group of wild type mice (WT IRI), sham operation group of S100A16^+/−^ mice (S100A16^+/−^ sham), ischemia–reperfusion injury (IRI) operation group of S100A16^+/−^ mice (S100A16^+/−^ IRI). The experimental protocol of IRI surgery were as follows: mice were intraperitoneally injected with 1.25% avertin (0.1 ml/5 g) (Sigma-Aldrich, St Louis, MO, USA) and placed on an electric blanket to maintain body temperature. Bilateral renal pedicles in mice were clamped for 30 min using micro-artery clips. Mice were euthanized after renal perfusion of 24 h, and then serum and kidney tissues were collected respectively. Serum creatinine level and BUN level were measured with an automated biochemical analyzer (7600-DDP-ISE; Hitachi Software Engineering, Yokohama, Japan) by Sir run hospital, Nanjing medical university (Nanjing, China). Part of each kidney was stored for histologic, IHC and IF staining, the left part of each kidney was extracted for mRNA and protein detection.

### Histology and immunohistochemical staining

The kidney tissues of mice were fixed in 4% paraformaldehyde overnight, gradient dehydrated, embedded in paraffin, and then sectioned into slices of about 5 μm thickness for HE staining, PAS staining, and IHC staining. The kidney sections were subjected to HE staining and PAS staining by routine protocols to detect renal histopathological changes. The PAS staining reagent was from Solarbio (Beijing, China). Tubular injury score after IRI was determined by PAS-stained sections according to the criteria: tubular dilation, brush border loss, tubular necrosis, and neutrophil infiltration in randomly chosen 10 high-power fields. Each field was scored from 0 to 5 (0 = normal; 1 = mild injury, 0–10%; 2 = moderate, 11–25%; 3 = severe, 26–49%; 4 = very severe, 50–75%; 5 = extensive injury, > 75% [52]. For IHC staining, the slices were placed in 0.01 M citrate buffer (pH 6.0) in a pressure cooker (95 °C, 2 min) to repair antigens, washed in phosphate-buffered saline (PBS), and then incubated with primary antibody overnight at 4 °C. After 5 min × 3 washes with PBS, the sections were incubated with the IHC secondary antibody (ZC-G2129; ZCIBIO, Shanghai, China) at 37 °C for 30 min. The sections were stained by 3,3′-Diaminobenzidine (DAB) reaction, counterstained with hematoxylin, routinely dehydrated, and then sealed with neutral glue. The primary antibodies against S100A16 (HPA045841; Sigma-Aldrich, St Louis, MO, USA), β-catenin (610153; BD Biosciences, San Jose, CA, USA), HRD1 (13473-1-AP; Proteintech, Chicago, IL, USA) were used with 1:100 dilution. All images were captured by using a digital microscope (IX51 + DP72; OLYMPUS, Tokyo, Japan). All operations were completed in the Sir run hospital, Nanjing medical university (Nanjing, China).

### Cell culture

Normal rat kidney fibroblasts (NRK-49F) cells were cultured in DMEM/F12 (Gibco, CA, USA) containing 10% fetal bovine serum (BI, Israel) at 37 °C with 5% CO_2_/95% air in a humidified incubator. NRK-49F cells are the gifts from Dr. Chunsun Dai (Center for Kidney Diseases, 2nd Affiliated Hospital, Nanjing Medical University; Nanjing, China).

### In vitro hypoxia/reoxygenation assay and ICG-001 treatment

NRK-49F cells were subjected to hypoxia/reoxygenation (H/R) to induce the IRI model in vitro. NRK-49F cells reached 70–80% confluence under normal atmosphere (5% CO_2_/95%), then growth-arrested in a serum-free medium for 16 h. Then NRK-49F cells were pretreated with 0, 5 or 10 μM of Wnt/β-catenin signaling pathway small molecule inhibitor ICG-001 (APExBIO, Houston, TX, USA, diluted in dimethyl sulfoxide) for 0.5 h, followed by hypoxia/reoxygenation experimentation. Hypoxia 24 h and reoxygenation 24 h in NRK-49F cells were established with Anaeropack (Mitsubishi gas chemical company, Tokyo, Japan) work as oxygen absorber-CO_2_ generator. The sachet of Anaeropack absorbed O_2_, generated about 16% of CO_2_ or more simultaneously, and produced an anaerobic atmosphere. The color of the oxygen indicator turned from pink to purple when the O_2_ concentration became less than 0.1%, and the O_2_ concentration changed to 0% after about 2 h. NRK-49F cells were incubated in anaerobic conditions for 24 h, then were transferred to a normal atmosphere for 24 h. Then NRK-49F cells were collected for western blot and qPCR assay.

### Transient transfections of plasmids, shRNA and adenovirus

The constructs of S100A16 OE, pcDNA3.1 (control plasmids of S100A16 OE), S100A16 shRNA, and scrambled shRNA (control S100A16 shRNA), were the gifts of Dr. Yun Liu (The First Affiliated Hospital of Nanjing Medical University) [25]. For S100A16 overexpressing, NRK-49F cells pretreated by 10 μM ICG-001 were transiently transfected with S100A16 OE or pcDNA3.1 by using lipofectamine 3000 reagent (Invitrogen Waltham, MA, USA) at 70–80% confluence. NRK-49F cells were then collected for western blot after transfection 48 h. For S100A16 knockdown, NRK-49F cells were transfected with S100A16 shRNA or scrambled shRNA using lipofectamine 3000 reagent at 70–80% confluence, and then performed with hypoxia/reoxygenation assay. Adenovirus HRD1 was purchased from Genechem (Shanghai, China). After transiently transfected with plasmids, NRK-49F cells were infected with adenovirus HRD1 for 48 h, and then harvested for western blot, qPCR and IF assay.

### Western blot

The kidney tissues of mice or treated NRK-49F cells were lysed with radioimmunoprecipitation assay buffer (RIPA buffer) containing 100 mg/ml phenylmethanesulfonyl fluorid (PMSF), and then subjected to 8%, 10% or 12% sodium dodecyl sulphate-polyacrylamide gel electrophoresis (SDS-PAGE) according to the molecular weights of target genes, transferred to polyvinylidene difluoride (PVDF) membrane, blocked with TBST buffer (1 × Tris-buffered saline, 0.1% Tween20 detergent) containing 5% nonfat milk for 2 h at room temperature. Primary antibodies were added to the blots at 4 °C overnight. The blots were washed with TBST × 3 times for 10 min each time, incubated for 1 h with horseradish peroxidase-conjugated secondary antibodies (31340, 31431; Thermo Fisher Scientific, Ann Arbor, MI, USA) in TBST at room temperature, and then washed as described above. The primary antibodies were used as followed: anti-S100A16 (11456-1-AP), anti-HRD1 (13473-1-AP), anti-GAPDH (60004-1-lg), and anti-β-actin (60008-1-lg) were purchased from Proteintech (Chicago, IL,USA), anti-Active β-catenin (8814S), anti-GSK3β (9832S), anti-BAX (2772S), anti-Cleaved caspase3 (9664S), anti-Caspase3 (9662S), and anti-Ubiquitin (3933S) were acquired from Cell Signaling Technology (Danvers, MA, USA), anti-β-catenin was purchased from BD Biosciences (610153; San Jose, CA, USA), anti-CK1α was obtained from Abcam (ab206652; Cambridge, UK), anti-HGF was purchased from Santa Cruz Biotechnology (sc-7949; Dallas, TX, USA). The band signals were visualized with the enhanced chemi-luminescence (ECL) Plus detection reagent (Thermo Fisher Scientific, Ann Arbor, MI, USA) and imaged with the Image Quant ECL system (PerkinElmer Life Sciences, Wellesley, MA, USA). Image Lab software (Bio-Rad, Hercules, CA, USA) was used to quantify the bands of western blot. The primary antibodies dilution for western blot is 1:1000. The secondary antibodies were diluted in 1:8000. The mouse samples were loaded with 100 μg, and the cell samples were loaded with 10 μg.

### Immunofluorescence staining

Mouse kidney sections or treated NRK-49F cells on slides were fixed in 4% paraformaldehyde solution at 4 °C overnight, and then 0.2% Triton X-100 was applied for permeabilization of the cell membrane. After 15 min permeabilization, the slides were blocked by 5% bull serum albumin (BSA) at 37 °C for 2 h. The slides were incubated with primary antibody at 37 °C for 2 h, and then washed by PBS at room temperature for 3 times × 5 min. Then the slides were incubated with the fluorescein isothiocyanate (FITC)-conjugated secondary antibody (A21203, A21206; Invitrogen, Waltham, MA, USA) at 37 °C for 1 h. After washing with PBS, the slides were stained by 4′6-diamidino-2-phenylindole (DAPI, D9542-10MG; Sigma-Aldrich, St Louis, MO, USA) for 2 min, and observed with a microscope equipped with a digital camera (IX51 + DP72; OLYMPUS, Tokyo, Japan) after washing. The antibodies against S100A16 (11456-1-AP; Proteintech, Chicago, IL, USA), PDGFRβ (sc-374573; Santa Cruz Biotechnology, Dallas, TX, USA), β-catenin (610153; BD Biosciences, San Jose, CA, USA), HRD1 (13473-1-AP; 67488-1-Ig; Proteintech, Chicago, IL, USA), GSK3β (9832S; Cell Signaling Technology, Danvers, MA, USA), CK1α (ab206652; Abcam, Cambridge, UK), were used with 1:100 dilution. The FITC-conjugated secondary antibodies were diluted in 1:1000, and the DAPI was diluted in 1:10,000.

### RNA extraction and real-time PCR

The kidney tissues of mice or treated NRK-49F cells were lysed by Trizol reagent according to the manufacturer’s instruction (Thermo Fisher Scientific, Ann Arbor, MI, USA). The cDNA was synthesized form RNA by reverse transcription in accordance with the reverse transcription kit system (TOYOBO, Osaka, Japan). PCR was performed by using SYBR Green Master Mix (Applied Biosystems, Foster, MI, USA) and real-time PCR system (Applied Biosystems Step OnePlus™, San Francisco, CA, USA). The mRNA expression of various target genes was normalized to the housekeeping gene β-actin, and the relative mRNA level compared to the control result was calculated by using the 2^−ΔΔCT^strategy. Primer sequences used for Real-time PCR were shown in supplementary material Table 1.

### Co-immunoprecipitation

The NP40 lysis buffer (50 mM Tris, 150 mM NaCl, 1 mM EDTA, 0.5% NP-40, 1 mM PMSF) was added to the cells, and then the cells were fully lysed on ice. The corresponding primary antibody against HRD1 (13473-1-AP; Proteintech, Chicago, IL, USA), GSK3β (9832S; Cell Signaling Technology, Danvers, MA, USA), or CK1α (ab206652; Abcam, Cambridge, UK) was added to about 500 µg cell proteins respectively, and then protein-antibody complex was incubated at 4 °C, 70 rpm (revolutions per minute) overnight. The 100ul washed protein A/G agarose beads (Thermo Fisher Scientific, Ann Arbor, MI, USA) were added to per protein-antibody complex, and then protein-beads complex was incubated at 70 rpm for 6 h at 4 °C. The complex was washed three times with NP40 lysis buffer, and then was added with 2 × SDS loading buffer (1 M Tris–HCl, 10% SDS, 50% glycerin, 0.5% bromophenol blue, and 5% β-mercaptoethanol). After placed at 95 °C for 10 min, the immunoprecipitated proteins were eluted, and then detected by immunoblotting. Controls were conducted by using IgG antibodies (YP0067, YP0068; YIFEIXUE Biotechnology, Nanjing, China) at amounts equal to the primary antibodies. The primary antibodies were diluted with 1:50.

### MG132 proteasome inhibition test

The NRK-49F cells were subjected to HRD1 adenovirus infection for 48 h, then the proteasome inhibitor MG132 (Enzo Life Science, Ann Arbor, MI, USA) was added to the NRK-49F cells wit h afinal concentration of 20 µM. After 6 h, NRK-49F cells were harvested for western blot analysis.

### Ubiquitylation assay

Ubiquitin-WT plasmids (Addgene, Watertown, MA, USA) were transfected with lipofectamine 3000 reagent in NRK-49F cells, and then the cells were transfected by adenovirus HRD1. After transfection for 24 h, the proteasome inhibitor MG132 was added to the cells at a final concentration of 20 µM for 6 h. Then cells were lysed in NP40 lysis buffer. The lysates were centrifuged to gain cytoplasmic proteins and incubated with anti-GSK3β antibody (9832S; Cell Signaling Technology, Danvers, MA, USA) or anti-CK1α antibody (ab206652; Abcam, Cambridge, UK) overnight, then mixed with protein A/G agarose beads at 4 °C for 6 h. After washing three times with NP40 lysis buffer, the beads released proteins by boiling them in 2 × SDS loading buffer. The released proteins were analyzed by western blot with anti-ubiquitin antibody (3933S; Cell Signaling Technology, Danvers, MA, USA) in 1:1000 dilution.

### Cycloheximide chase assay

NRK-49F cells were infected with HRD1 adenovirus to induce HRD1 overexpression. 48 h later, cycloheximide (Sigma-Aldrich, St Louis, MO, USA) was added at a final concentration of 100 μg/ml (diluted in dimethyl sulfoxide) in cells, and then the cell proteins were harvested at different time points (0, 2, 4, 6 h) after the addition of cycloheximide. SDS-PAGE method as described above.

### Statistical analysis

All data were presented as mean ± SEM. Statistical analyses of the data were performed using statistical analysis software GraphPad Prism 8.0.2 (GraphPad Software, Inc., La Jolla, CA, USA). Comparison between cell groups was made using one-way ANOVA followed by Fisher’s Least Significant Difference (LSD) test. Comparison between animal experiment groups was made using two-way ANOVA followed by Tukey test. *P* < 0.05 was considered statistically significant.

## Supplementary Information

Below is the link to the electronic supplementary material.

Supplementary file1 (PDF 1451 KB)

## Data Availability

All data generated during this study are included in this published article and its supplementary information files.
