# Supplementary material for: S100A16 promotes acute kidney injury by activating HRD1-induced ubiquitination and degradation of GSK3β and CK1α
Source: Cell Mol Life Sci. 2022 Mar 12;79(3):184. doi: 10.1007/s00018-022-04213-5 (PMC8918193; doi:10.1007/s00018-022-04213-5)
Supplement: Supplementary file 1 — Supplementary file1 (PDF 1451 KB) [file 18_2022_4213_MOESM1_ESM.pdf]

Supplementary Data

Title: S100A16 promotes acute kidney injury by activating HRD1-induced ubiquitination and degradation of GSK3β and CK1α

Authors:

Yifei Sun[1], Ya Fan[2], Zheng Wang[1], Min Li[2], Dongming Su[2], Yun Liu[3] and Xiubin Liang[1*]

1. Department of Pathophysiology, Nanjing Medical University, Nanjing 211166, China
2. Department of Pathology, Nanjing Medical University, Nanjing 211166, China.
3. Department of Geratology, The First Affiliated Hospital of Nanjing Medical University, Nanjing 210029, China

*Address correspondence:

Xiubin Liang

Department of Pathophysiology,

Nanjing Medical University,

Nanjing, Jiangsu Province,

China. 211166

Email: liangxiubin@njmu.edu.cn

Figure S1. Renal tubular injury score and mRNA levels of Wnt ligands in AKI mice.

(a) Tubular injury score in WT mice and S100A16$^{+/-}$ mice after IRI. **** *P*<0.0001, 10 high-power fields in every group were chosen. (b-i) Real-time qPCR analysis showed different magnitudes of Wnt induction after IRI. Knockout of S100A16 suppressed the increased mRNA expressions of Wnt genes relative to β-actin in the kidneys at 1 day after IRI. **** *P*<0.0001, *** *P*<0.001, n.s. not significant. n=3; each point represents the expression in a sample pooled from two mice.

Figure S1

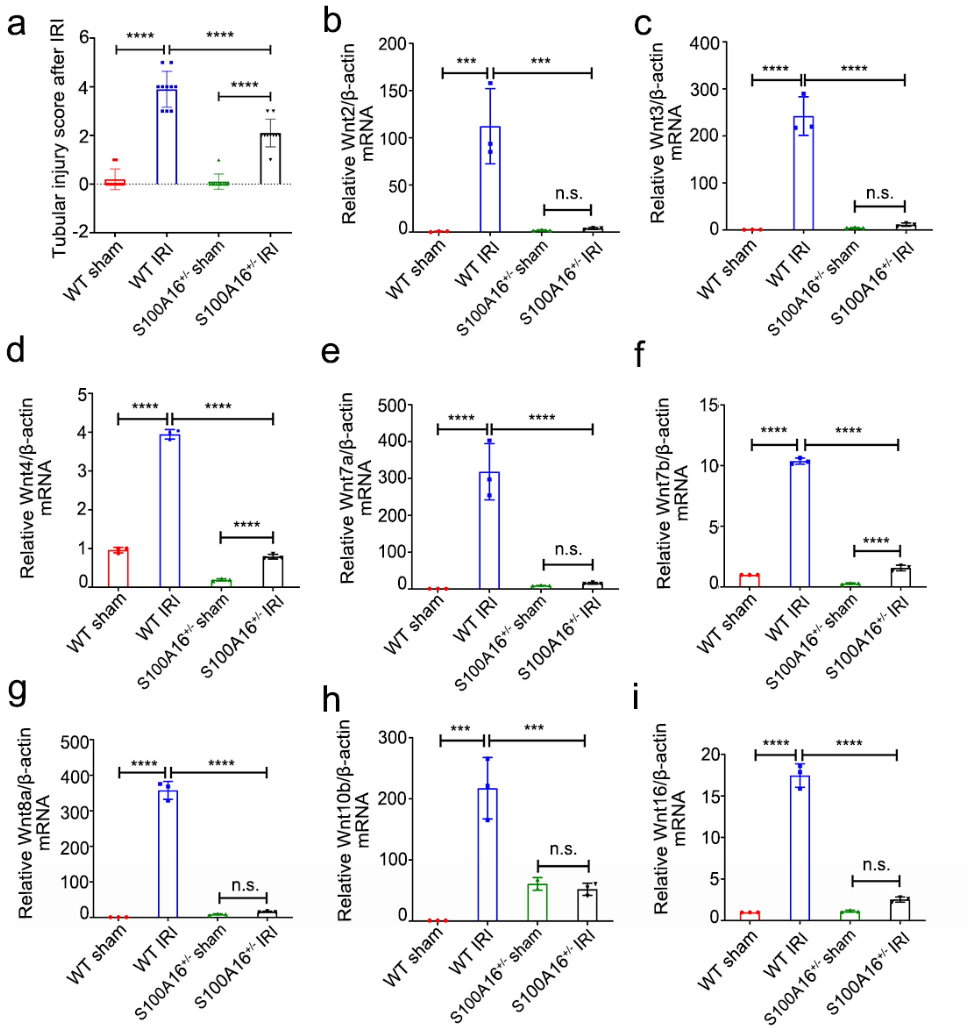

Figure S2. S100A16 is localized in PDGFRβ positive renal fibroblasts.

Double immunofluorescence staining in the corticomedullary junction of mice kidneys showed the localization of S100A16 and PDGFRβ at 1 day after IRI and compared it with sham mice. White arrows indicate the co-localization of S100A16 and PDGFRβ in renal fibroblasts. Scale bar, 20μm.

Figure S2

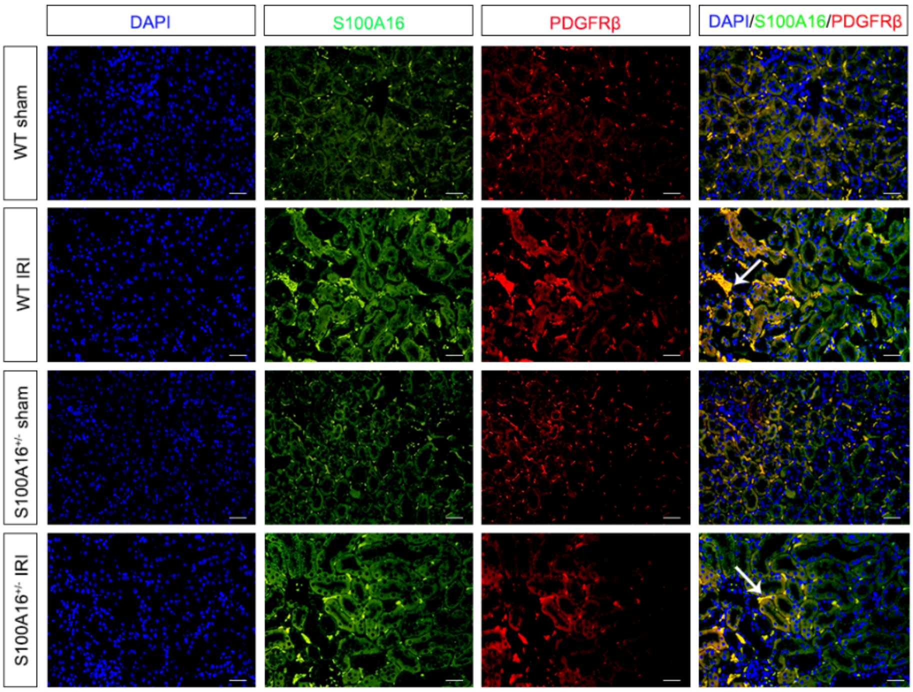

Figure S3. HRD1 mRNA level is increased in the kidneys of AKI mice and in hypoxic renal fibroblasts.

(a) The relative HRD1/β-actin mRNA expression were tested using kidney tissues from WT mice and S100A16$^{+/-}$ mice at 1 day after IRI compared with sham mice using real-time qPCR analysis. *** $P<0.001$, ** $P<0.01$, * $P<0.05$. n=3; each point represents the expression in a sample pooled from two mice. (b) Real-time qPCR analysis showed elevated relative HRD1/β-actin mRNA level in NRK-49F cells treated with H/R assay. ** $P<0.01$. n=3.

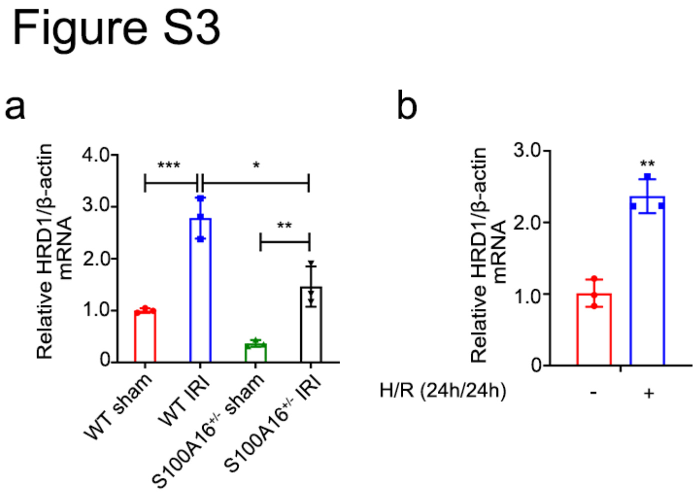

Figure S4. HRD1 and GSK3β or CK1α partially colocalize in NRK-49F cells.

(a) HRD1 and GSK3β partially colocalize in the cytoplasm of NRK-49F cells. Scale bars, 20μm. (b) HRD1 and CK1α partially colocalize in the cytoplasm of NRK-49F cells. Scale bars, 20μm.

Figure S4

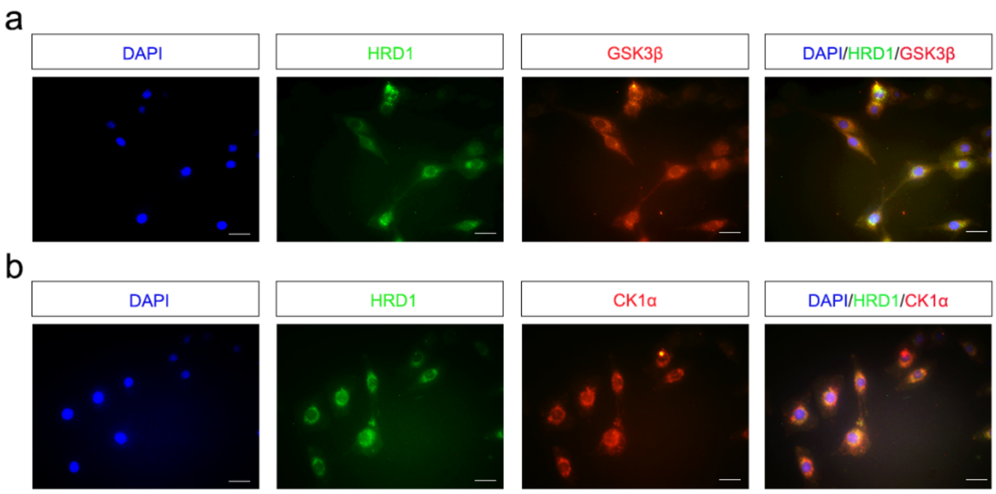

Figure S5. HRD1 does not affect GSK3β or CK1α expression at the post-translational level.

(a) The mRNA level of GSK3β relative to β-actin showed no significant differences in the presence or absence of HRD1 overexpression in NRK-49F cells. n.s. no significance. n=3. (b) The relative CK1α/β-actin mRNA level exhibited no significant differences in the presence or absence of HRD1 overexpression in NRK-49F cells. n.s. no significance. n=3.

Figure S5

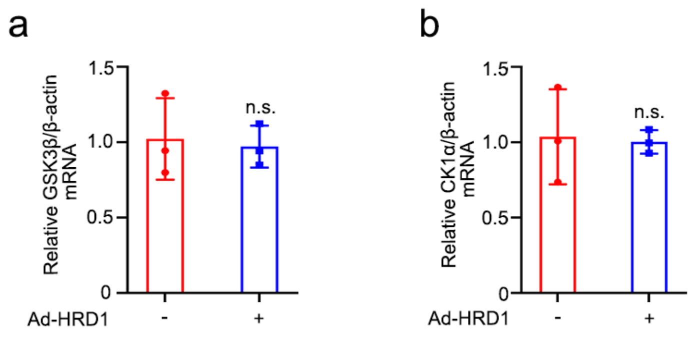

Table 1. Nucleotide sequences of the primers for Real-time PCR.

Gene	Forward (5'-3')	Reverse (3'-5')
Wnt2	AACGTCCCTCTCGGTGGAATC	TGTACCACCATGAAGAGCTGACC
Wnt3	CAGCGTAGCAGAAGGTGTGAAG	ATGGCCAGGCTGTCATCTATG
Wnt4	GCTGTACCTGGCCAAGCTGTC	TGGATCAGGCCTTTGAGTTTCTC
Wnt7a	TGCGTGCCAGTCGAAACAAG	GATATACACCAGGTCAGTGTCCATGG
Wnt7b	GCCAACATCATCTGCAACAAGA	CCGATCACAATGATGGCATC
Wnt8a	CAGCGACAACGTGGAGTTCG	CATCCTTCCCTTTCTCCAAACTG
Wnt10b	ACGACATGGACTTCGGAGAGAAGT	CATTCTCGCCTGGATGTCCC
Wnt16	ACCCCCATTCTCAAGGATGACTT	CAGTTTCTTGTTCTCCACGCAGTA
HRD1	AACCCCTGGGACAACAAGG	GCGAGACATGATGGCATCTG
GSK3β	CACAGAGCAGCTCCTGACC	CTCCTCGCTTCCTTCCTTCC
CK1α	CCAAACCCCCACAGGTTTCTAA	ATGCTGCCCAGAGTCAGTTT
HGF	TTTCCCGTTGTGAAGGAGATAC	ATTTCAAACTAACCATCCACCC
β-actin	GGCTGTATTCCCCTCCATCC	CCAGTTGGTAACAATGCCATG
